# Establishing a veterinary anatomy core syllabus through a modified Delphi process

**DOI:** 10.1111/joa.13948

**Published:** 2023-09-04

**Authors:** Erica Gummery, Miren Singh, Sarah B. Channon

**Affiliations:** ^1^ School of Veterinary Medicine and Science University of Nottingham Sutton Bonnington UK; ^2^ Department of Comparative Biomedical Sciences Royal Veterinary College, University of London London UK

**Keywords:** anatomy knowledge, consensus survey, curriculum, education, veterinarian

## Abstract

Anatomy forms a key component of veterinary curricula, but, in the context of an evolving profession, curricula are adapting and changing accordingly. There is a lack of guidance for educators regarding the levels of anatomical knowledge required for a graduate to be considered safe or competent. A formal review of veterinary anatomy learning outcomes (LOs) is therefore timely to support curriculum development in this rapidly evolving field. This study aimed to create a set of LOs which reflect the recommended core requirements for a new graduate veterinarian. A consensus approach using a modified Delphi method was used. The Delphi panel consisted of 23 experienced and active veterinary anatomy educators from veterinary schools within the UK and Ireland. The process had four stages: (1) Research team review, pre‐screening and modification of a list of existing LOs (adapted from the Core Regional Anatomy Syllabus) which then formed the initial set of outcomes sent for review; (2) Delphi Round 1; (3) Delphi Round 2; (4) Post‐Delphi final screening and review. Qualitative data outlining the rationale for modification and rejection of LOs were analysed via content analysis. 167 LOs were initially presented to the Delphi panel in Round 1. 64 of those were accepted, 79 recommended for modification and 23 rejected. 122 LOs were presented to the Delphi panel in Round 2. Of these, 86 outcomes were accepted, 10 modified and 26 rejected. 160 LOs were ultimately accepted and form the Veterinary Anatomy Core Syllabus. Key themes arising from analysis include the removal of unnecessary detail and increased focus on the relevance of competencies required of a new veterinary graduate. The syllabus presented may be used by curriculum planners, teachers and students within veterinary education worldwide.

## Graphical abstract

This study used a modified Delphi approach to establish recommended core anatomy knowledge for a new graduate veterinarian. 160 learning outcomes form the Veterinary Anatomy Core Syllabus which may be used by curriculum planners, teachers and students within veterinary education worldwide. Recommendations of Delphi panellists included the removal of unnecessary detail and increased focus on the relevance of competencies required of a new veterinary graduate.

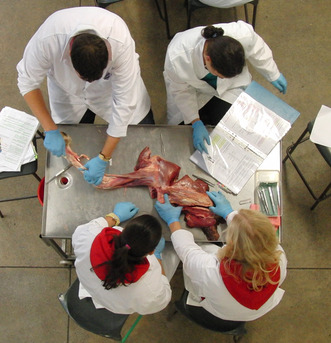

## 1 INTRODUCTION

Anatomy forms a fundamental component of medical, veterinary and healthcare professions alike. It is crucial for performing basic physical examinations, communicating with clients and colleagues, understanding normal function, as well as comparing normal with abnormal clinical presentations. The development of sophisticated diagnostic imaging modalities has further increased the requirement for good anatomical knowledge amongst veterinary practitioners, especially in equine veterinary practice (Homfray et al., 2022). Further, anatomical knowledge underpins the successful and safe conduct of more complicated clinical procedures and surgeries. To date there is insufficient formal research into what levels of anatomical knowledge are required for a graduate to be considered ‘safe’. In the context of veterinary education, producing graduates who are competent and safe is of particular importance as unlike their medical equivalents who enter a supervised Foundation Programme, a newly qualified veterinary surgeon can register to practice medicine and perform surgery unsupervised from the day on which their degree is conferred. Therefore, a comprehensive foundation of key anatomical knowledge is imperative for the safety and welfare of their patients. Furthermore, uncertainty in anatomical knowledge impacts confidence and decision‐making in veterinarians working within clinical practice (Wheble & Channon, 2021), highlighting the potential impacts of failing to adequately prepare students in this subject on the individual veterinarians and the profession, as well as the patients.

Echoing medical curricula, newly developed and recently reviewed programmes have moved to a more outcomes‐based approach to veterinary education, and a more integrated approach ‘where the clinical and basic sciences are taught and learned together’ (General Medical Council, 2016). Like medical programmes, veterinary programmes have seen a squeeze on teaching time dedicated to anatomy and other sciences, as curriculum space has been reapportioned to allow sufficient focus on professional and clinical skills to ensure that students are ready for the evolving role of a veterinary professional (Lane et al., 2017; Parkinson et al., 2017; Radostits, 2003).

A focus on the attributes of a veterinary professional more holistically, coupled with the wide‐ranging career choices available to a new graduate veterinary surgeon has led to a lack of specificity with regard to anatomical knowledge in the Royal College of Veterinary Surgeons (RCVS) list of Day One Competences (The Royal College of Veterinary Surveons, 2022). Further, a veterinary student graduates as omnicompetent and able to treat all species, creating further challenges in defining what is appropriate for ‘day one’. For example, a detailed understanding of equine limb anatomy is vitally important for equine practice, with musculoskeletal conditions presenting as the most common condition in horses (Nielsen et al., 2014). However, many more new veterinary graduates choose to work in small animal practice than in equine practice (56.4% and 6.3% respectively (The Royal College of Veterinary Surgeons, 2021)), and a much less detailed knowledge of homologous structures is required in a small animal setting. There is continued discussion regarding species specialisation, or tracking, within the veterinary curriculum, but accrediting bodies of veterinary curricula currently require training in all species (American Veterinary Medical Association, 2021; The Royal College of Veterinary Surgeons, 2022). Therefore, an outcomes‐based anatomy curriculum needs to reflect the requirements of these professional bodies.

Whilst undoubtedly important, anatomy teaching can be challenging. The principal difficulties reported by educators include the vastness of the content (both in breadth and depth), and how to successfully convey or deliver this content to students and learners (Hall et al., 2018; Javaid et al., 2018). Three main barriers to learning anatomy have been suggested: visualisation of structures, the extensive body of information and inherent issues with curriculum design (Cheung et al., 2021). Alongside these challenges, the low availability of curriculum time for teaching gross anatomy, a shortage of trained anatomists and an increased cost associated with obtaining cadaveric specimens have been identified as further issues for educators (Naidoo et al., 2020). Additionally, the COVID‐19 pandemic has had considerable effects on anatomy education, as novel and innovative methods of teaching have had to be implemented. This includes the use of online resources, videos, increased use of prosections, 3D printing and virtual reality (Iwanaga et al., 2021). It will be interesting to see the continued evolution of anatomy teaching in the upcoming years, and how the various challenges will be addressed for future veterinary, medical and allied health sciences graduates.

Due to a previous lack of a well‐defined anatomy curriculum in the field of human medicine (despite numerous attempts) (Keers et al., 2000; Kilroy & Driscoll, 2006; Leonard, 1996), the Anatomical Society created a syllabus in order to display the minimum requirements suggested for a newly qualified medic (McHanwell et al, 2007), later revised and published as the Core Regional Anatomy Syllabus (CRAS) (Smith, Finn, Stewart, Lee, et al., 2015; Smith, Finn, Stewart, & McHanwell, 2015). Though the long‐term impact of CRAS on anatomical teaching is yet to be studied, initial evaluations suggest that the revised core syllabus has helped cultivate professional identity and support networks amongst anatomists (Smith et al., 2019). The CRAS has helped anatomists provide a particular level of course content that can give graduates confidence as practitioners through a deeper understanding of what knowledge is required (Smith et al., 2019; Young, 2007). Such is the perceived strength and usefulness of the core syllabus approach, many further syllabi have since been developed to support and provide an evidence base for anatomy teaching within other healthcare fields, including nursing (Connolly et al., 2018), pharmacy (Finn et al., 2018), and dentistry (Matthan et al., 2020).

The purpose of this current study was to add to the series of core anatomy syllabi by developing a core syllabus for veterinary anatomy. This will provide the necessary guidance for veterinary anatomy educators and those developing veterinary medicine, surgery and science programmes. To achieve this, this study specifically aimed to determine the core foundational anatomical knowledge needed by new graduate veterinarians, through a modified Delphi consensus approach.

## 2 MATERIALS AND METHODS

This study was approved by both the Social Sciences Research Ethics Review Board at the Royal Veterinary College (ID URN 2020 0125) and the Committee for Animal Research and Ethics at the University of Nottingham (ID 3221 200817).

A consensus approach using a modified Delphi method (as described in Smith, Finn, Stewart, & McHanwell, 2015) was used to derive a syllabus for Veterinary Anatomy, consisting of a set of agreed learning outcomes (LOs). Modified Delphi approaches use an existing list of LOs as a starting point for amendment by a Delphi panel. The process had four stages: (1) Research team review, pre‐screening and modification of existing LOs; (2) Delphi Round 1; (3) Delphi Round 2 and (4) Post‐Delphi final screening and review.

### 2.1 Development of an initial list of learning outcomes

The initial list of LOs for this study was created by combining outcomes previously published for human anatomy within a medical curriculum (Smith, Finn, Stewart, Lee, et al., 2015) with a sample of neuroanatomy LOs from a published core syllabus for the teaching of neuroanatomy to medical students (Moxham et al., 2015). It was decided to add these selected neuroanatomy LOs, since the Anatomical Society Core Regional Anatomy Syllabus (Smith, Finn, Stewart, Lee, et al., 2015) opted to exclude neuroanatomy, however this subject is taught as an important part of integrated veterinary anatomy curricula at many veterinary schools. The selection and subsequent editing of the initial set of LOs was undertaken by the research team (authors of this study, two veterinary anatomy educators from different UK veterinary schools and a veterinary medicine graduate working in first opinion veterinary practice in the UK). Several steps were required to edit the combined initial list of LOs to make them suitable for a veterinary anatomy context. Most UK veterinary schools teach veterinary anatomy on a body system, rather than a regional, basis therefore the initial list of LOs was reorganised to reflect a body systems approach. LOs were pre‐screened for relevance to veterinary medicine and any which were clearly not were removed by the research team at an early stage (e.g. ‘Describe the main types of grip (power, precision and hook) and the role of the muscles and nerves involved in executing them’), though, if there was any doubt these were left within the list for the Delphi panel to later consider. Following this, the research team screened the list of outcomes for human‐centric terminology, editing accordingly for a veterinary emphasis. For example, directional terms such as anterior and posterior were replaced with suitable equivalents (e.g. ventral and dorsal), and human related anatomical terms (e.g. ‘hand’) were revised accordingly.

Species relevance of each of the LOs was considered. Points of clarification such as ‘common veterinary species’ or ‘ruminants’, were incorporated where considered appropriate. In some instances, the research team felt it necessary to introduce new additional LOs, where it was felt the final list of LOs omitted key species‐specific structures or adaptations of importance. However, for the most part, since our objective was to create a syllabus that could be utilised by veterinary anatomy educators globally, we refrained from being overly specific in relation to species, mindful that cultural and practical differences will influence which species are considered ‘key’ and of importance at different institutions.

The research team derived basic rules regarding the degree to which related, integrated and applied topics such as imaging, topographic anatomy, pathology, histology, embryology and physiology should be reflected in the final list of LOs. To ensure the list of LOs remained manageable in length we elected to include only one generic imaging related LO and one topographical anatomy outcome for each body system at the initial stage. We elected to avoid direct reference to histology, pathology and embryology, and to refer to physiology only in so much detail as to expect a functional understanding of anatomical structures.

Finally, the list of LOs was reviewed by the research team for any duplication, clarity of phrasing and typographic considerations before being sent to the Delphi panel for review. The initial list of outcomes for review by the Delphi panel consisted of 167 statements.

### 2.2 The Delphi panel

A Delphi panel was identified by the research team and invited to participate in this study. To be invited to participate panellists needed to be either a veterinarian involved in undergraduate veterinary anatomy education or an anatomist working in veterinary education. Panel participants were required to be an Associate Fellow, Fellow, Senior Fellow or Principal Fellow of Advance HE (formerly HEA) or to have a minimum of 3 years of experience in their current role. A list of potential panel members who fulfilled these criteria was created by the research team and these individuals were recruited directly, via email. Additionally, expressions of interest were sought beyond this group through an advertisement in Anastomosis, the newsletter of the Anatomical Society and through an email distribution to heads of eight UK veterinary schools via the Veterinary Schools Council.

### 2.3 The Delphi process

An online survey (JISC surveys) was used to allow participants to view the LOs and participate in the Delphi process. The survey contained questions to ascertain basic demographic information of the panellists, as well as the initial list of LOs. These were presented with options for panellists to either accept in their current form, reject or to modify (with free text comment boxes provided for suggested modifications). Optional open text boxes were also provided at the end of each body system‐based section to allow panellists to make general comments or to list any new LOs they felt should be added. The survey was piloted internally at the institutions of the research team before wider circulation, and as a result minor typographical amendments were made. Individuals completing the survey as a pilot were not included in the Delphi panel. Consensus for agreement for both Round 1 and 2 of the Delphi process was set as 90%—this was a minimum value, and for the most part consensus was reached at a higher level.

Round 1 of the Delphi process was open to participants for a 1‐month period from 17 May to 17 June 2021. 23 individuals participated in Round 1. Following closure of the survey, where the consensus level for LOs were met, these were automatically accepted for inclusion into the syllabus. Where the consensus level was not met, the data were reviewed by the research team who used the following protocol for decision making:

- Where the majority of respondents suggested a particular modification, this change was made.
- Where different or contradictory modifications were suggested for a particular LO, the changes made were decided through discussions between members of the research team. The decisions made were based on ensuring a balanced perspective, ensuring consistency and avoiding repetition within the syllabus. The rationale for decisions was documented.
- Where the majority of respondents suggested rejection of a LO, this was removed from the syllabus.
- When respondents were split between rejecting or modifying a LO, a final decision was made based on the comments and suggested modifications of the participants. If it was felt that the suggested modifications were not appropriate, or comments suggested compelling reasons for rejection or noted repetition, the outcome was removed.

Any new LOs suggested by participants were reviewed by the research team to ensure they met the basic rules for inclusion that were employed when drawing up the initial list of LOs. New outcomes deemed appropriate were included for review in Round 2 of the process, together with modified outcomes arising from Round 1. All new outcomes could be accepted, modified, or rejected by panellists. Previously modified outcomes were only available for panellists to either accept or reject. A downloadable .pdf document of all previously accepted LOs was provided for participants during Round 2 to ensure that participants had the necessary context and information to make decisions about content. Round 2 was open to participants for a 3‐week period from 8 to 30 September 2021. 14 individuals participated in Round 2. Following closure of the survey, where the consensus level for LOs were met, these were automatically accepted for inclusion into the syllabus. Where the consensus level was not met, the data were reviewed by the research team who followed the same protocol for decision making as for Round 1. Following Round 2, LOs were post‐screened to check for duplication, grammar, spelling and to ensure that they followed an appropriate sequence.

### 2.4 Data analysis

Quantitative data were compiled and analysed in Excel. Following cessation of the Delphi process, inductive coding of the qualitative data was carried out (NVivo), as described by Thomas (2006). Data were read, reflected upon and categorised. Following a process of revision and refinement lead to the creation of the final categories. Initial codes were agreed upon by two researchers before final coding and analysis took place. Quantitative data (% rates of acceptance) were aggregated by demographic characteristics of participants.

## 3 RESULTS

### 3.1 Respondents

**FIGURE 1:**
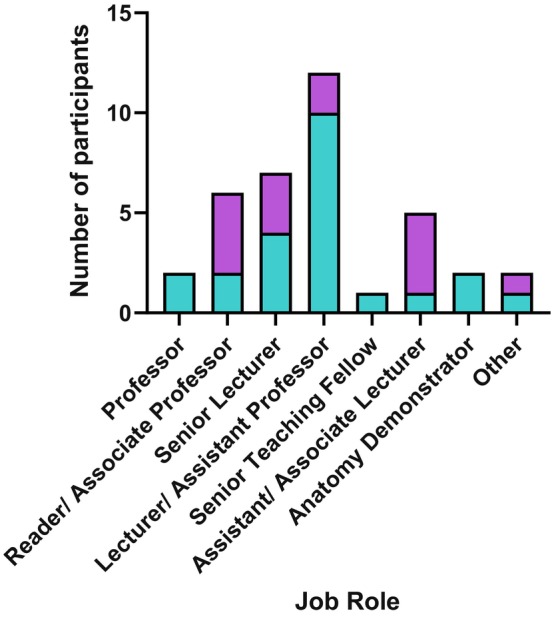
Academic role profile of consensus survey respondents during Delphi Round 1 (blue) and Round 2 (purple).

Of the 23 individuals who participated in Round 1, most were employed at Assistant Lecturer/Lecturer level (43%) with smaller numbers of individuals in other job roles (Figure 1). 100% of respondents were based within a university for their employment. Of the 14 participants in Round 2 there was a broader spread of job roles and slightly more senior profile represented (Figure 1).

### 3.2 Delphi round one

**FIGURE 2:**
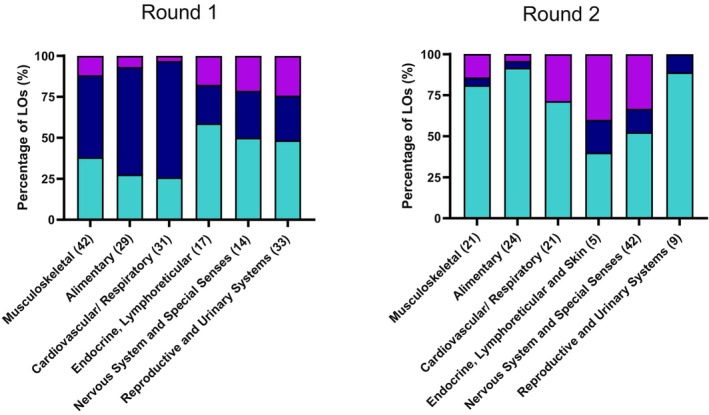
Percentage of Learning Outcomes accepted (light blue), modified (dark blue) and rejected (purple) for each round of the Delphi process, by body system section of the syllabus. Numbers in brackets indicate the number of learning outcomes that entered that round of the process for that body system.

Following Round 1 of the Delphi process, 64 outcomes were accepted, 79 recommended for modification and 23 rejected at this stage (Table 1). Considered by body system, the highest proportion of accepted LOs in this round was within the endocrine, lymphoreticular and skin section of the syllabus, with 59% of initial LOs accepted. By comparison within the cardiovascular and respiratory and alimentary sections only 26% and 28% of LOs were initially accepted respectively (Figure 2).

**TABLE 1:** Learning outcomes rejected by the Delphi panel during the process and reasons for their removal.

Section	Learning outcome	Stage	% accept/reject/modify	Reason(s) for removal
1. Alimentary system	Explain the nerve supply of the parietal and visceral peritoneum	R1	63.6/27.3/9.1	Did not meet threshold High number of requests to reject
	Describe the origin and course of the inferior vena cava and its major tributaries	R1	40.9/13.6/45.5	Did not meet threshold Repetition identified
	Recognise that sinuses and nasal cavities differ amongst veterinary species	R1	73.9/0/26.1	Did not meet threshold Comments suggest poor outcome & content covered elsewhere
	Describe the anatomical arrangement of the lymphoid tissue in the pharyngeal and nasal walls	R1	77.3/18.2/4.5	Did not meet threshold One modification suggestion incorporated elsewhere; majority wished to reject
	Identify the spleen ultrasonographically	R1	73.9/17.4/8.7	Did not meet threshold Not specific enough Incorporated in modification of different LO
	Describe the blood supply and venous drainage of the distal colon, and explain its clinical significance	R2	78.6/21.4/0	Did not meet threshold Modification not an option
2. Cardiovascular/Respiratory systems	Describe the hyoid bones and cartilages of the larynx including relationships of the thyroid bone to the larynx and skull. Recognise how laryngeal structures differ between species	R2	71.4/28.6/0	Did not meet threshold Modification not an option
	Describe the intrinsic and extrinsic laryngeal muscles responsible for closing the laryngeal inlet and controlling vocal fold position and tension. Explain the innervation of the functional muscle groups that widen and narrow the glottis and their clinical significance in common veterinary species	R2	85.7/14.3/0	Did not meet threshold Modification not an option
	Describe the contents of the cranial and caudal parts of the mediastinum	R2	85.7/14.3/0	Did not meet threshold No modifications suggested
	Briefly summarise the anatomy of the bronchial tree and bronchopulmonary segments in common veterinary species and explain their functional and clinical significance	R2	85.7/14.3/0	Did not meet threshold Modification not an option
	Describe the origin and course of the left and right coronary arteries and discuss the functional consequences of anomalies	R2	57.1/42.9/0	Did not meet threshold Modification not an option
	Describe the origins, courses and relationships of the cranial and caudal venae cavae in common veterinary species	R2	85.7/14.3/0	Did not meet threshold Modification not an option Repetition identified
3. Endocrine, lymphoreticular and skin systems	Describe the structure of a hair follicle	R2	78.6/21.4/0	Did not meet threshold Modification not an option
	Identify and describe the function of the uropygial gland in birds that possess it	R2	57.1/42.9/0	Did not meet threshold Modification not an option
	Describe the flow of lymph around the body.	R1	87/13/0	Did not meet threshold No modifications suggested
4. Musculoskeletal system	Describe the origin, course and distribution of the major arteries within the thoracic limb, in relation to common sites of injury. Identify those sites where neurovascular structures are at particular risk of damage from musculoskeletal injuries	R1	81.8/9.1/9.1	Did not meet threshold Repetition identified
	Describe the origin, course and function of the axillary, radial, musculocutaneous, median and ulnar nerves in the thoracic limb	R1	77.3/4.5/18.2	Did not meet threshold Repetition identified
	Demonstrate the origin, course and branches of the major arteries that supply the gluteal region, hip, thigh, leg, hock and distal limb. Explain the functional significance of anastomoses between branches of these arteries at the hip and stifle	R1	60.9/13/26.1	Did not meet threshold Repetition identified
	Demonstrate the course of the principal veins of the lower limb. Explain the role of the perforator veins between the superficial and deep veins and the function of the ‘muscle pump’ for venous return to the heart. Describe the surface landmarks for sites of venous access that can be used for ‘cut‐down’ procedures in emergencies	R1	63.6/9.1/27.3	Did not meet threshold Repetition identified so incorporated in similar LO
	Describe the close relations of the stifle joint, including synovial structures and explain the relevance of these to arthrocentesis	R1	82.6/17.4/0	Did not meet threshold Arthrocentesis not a day one competency
	Demonstrate the sites at which pulses of the brachial, radial, ulnar, and digital arteries may be located in common veterinary mammalian species	R2	85.7/14.3/0	Did not meet threshold Modification not an option
	Compare the anatomy and movements of the pectoral girdle in animals adapted for terrestrial locomotion and flight	R2	78.6/21.4/0	Did not meet threshold Modification not an option
	Describe the anatomy and the range of motion of the sacroiliac joint. Demonstrate the location of the sacrotuberous ligament in small animals and the sacrosciatic ligament in large animals	R2	78.6/21.4/0	Did not meet threshold Modification not an option
5. Nervous system and special sense organs	Describe the boundaries, walls and floors of the cranial fossae and the relationships between the structures of the brain and the rostral, middle and caudal cranial fossae	R1	73.9/26.1/0	Did not meet threshold No modifications suggested
	Describe the functional architecture of the cerebellar cortex (molecular, purkinje and granule cell layers)	R1	65.2/30.4/4.3	Did not meet threshold High number of requests to reject
	Describe the deep cerebellar (intracerebellar) nuclei—dentate, emboliform, globose and fastigial nuclei)	R1	39.1/52.2/8.7	Did not meet threshold High number of requests to reject
	Describe the arrangement of the pia, arachnoid, and dura mater within the cranial cavity and in relation to the brain	R2	78.6/21.4/0	Did not meet threshold Modification not an option
	Describe the white matter of the cerebellum—intrinsic, afferent and efferent fibres of cerebellar hemispheres, and the corticopontocerebellar pathway	R2	28.6/42.9/28.6	Did not meet threshold High number of requests to reject
	Describe the general sensory nuclei and motor nuclei of the cranial nerves (somatic motor, branchomotor, and general visceral nuclei)	R2	50/28.6/21.4	Did not meet threshold High number of requests to reject
	Describe the location and external features of the midbrain, to include the aqueduct, the crus cerebri, the substantia nigra, the red nucleus, the cranial and caudal colliculi, and cranial nerve nuclei III and IV	R2	28.6/57.1/14.3	Did not meet threshold High number of requests to reject
	Describe the components of basal ganglia (basal nuclei) and their regional anatomy. Describe the circuits between the basal ganglia and the cortex	R2	35.7/64.3/0	Did not meet threshold High number of requests to reject
	Describe the structure and function of the cerebral white matter, including commissures, association, and projection fibres	R2	46.2/53.8/0	Did not meet threshold High number of requests to reject
	Describe the general arrangement and functions of the reticular formation	R2	64.3/28.6/7.1	Did not meet threshold High number of requests to reject
	Describe the optic nerve and its central connections to include the optic chiasma, optic tract, lateral geniculate body, and geniculo‐calcarine tract	R2	64.3/21.4/14.3	Did not meet threshold Modifications suggested duplication, so elements included within separate modified LO
	Describe the intracranial and intrapetrous course of the facial nerve and the relationships of its major branches to the middle ear in relation to damage of the nerve within the facial canal	R2	78.6/14.3/7.1	Did not meet threshold Suggested modification not significantly different to original
	Identify basic neuroanatomical structures and regions on CT and MRI scans of the brain, and vertebral column/spinal cord	R2	78.6/21.4/0	Did not meet threshold Comments suggest not a day one skill
	Describe the anatomy of the spinal cord to include the basic structure of white matter in the spinal cord and the functional localisation of neurons in the ventral horn, the dorsal horn; the intermediolateral horn, the grey commissure and the central canal	R2	85.7/7.1/7.1	Did not meet threshold Repetition identified
	Describe the structure and function of the ascending/descending tracts in the spinal cord including the dorsal white column (fasciculi gracilis and cuneatus) the lateral white column (dorsal and ventral spinocerebellar tracts, lateral spinocerebellar tract, dorsolateral/Lissauer's tract, lateral corticospinal tract, rubrospinal tract), the ventral white column (ventral spinothalamic tract, ventral corticospinal tract, vestibulospinal tract)	R2	35.7/50/14.3	Did not meet threshold High number of requests to reject
	Describe the structure and function of the lateral and ventral spinothalamic tracts (ventrolateral system) with respect to pain and temperature pathways, and light touch and pressure pathways (including consideration of the effects of injury to the lateral/ventral spinothalamic tracts)	R2	85.7/14.3/0	Did not meet threshold No modifications suggested
	Describe the structure and function of the dorsal and ventral spinocerebellar tracts in relation to balance and coordination	R2	85.7/14.3/0	Did not meet threshold No modifications suggested
6. Reproductive and urinary systems	Describe the anatomy and neurovascular supply of the clitoris, vulva and vagina. Explain the anatomy of the urogenital diaphragm	R1	81.8/13.6/4.5	Did not meet threshold Questionable relevance for veterinary students
	Describe the location and function of nasal salt glands in avian species	R1	59.1/27.3/13.6	Did not meet threshold High number of requests to reject
	Describe the skeletal and ligamentous components of the pelvis, the anatomy of the pelvic inlet and outlet and recognise their normal orientation. Explain sexual and species differences in pelvic skeletal and muscular anatomy	R1	82.6/13/4.3	Did not meet threshold No modifications suggested
	Describe the anatomy and functional importance of the pelvic diaphragm, its midline raphe, perineal body, attachment points and the structures passing through it in males and females. Describe the clinical significance of the pelvic diaphragm, e.g. in relation to continence/prolapse.	R1	68.2/9.1/22.7	Did not meet threshold Repetition identified
	Describe the autonomic innervation of the urinary bladder	R1	87/8.7/4.3	Did not meet threshold Covered in another more general LO
	Interpret standard diagnostic images of the pelvis and perineum, e.g. CT, MRI, X‐ray and ultrasound, and be able to recognise common abnormalities	R1	87/0/13	Did not meet threshold Repetition identified
	Explain the relative locations of the kidneys and bladder in the abdomen	R1	82.6/8.7/8.7	Did not meet threshold Comments suggest content covered elsewhere
	Describe the position and anatomy of the kidneys in common veterinary species	R1	82.6/0/17.4	Did not meet threshold Comments suggested incorporating the species specific component into modification of an existing LO to minimise duplication.
	Describe the structure of the kidney in birds, reptiles and mammals	R1	82.6/8.7/8.7	Did not meet threshold Few modifications suggested, but outside the remit of the syllabus

438 suggestions for modification were made by participants. Review of the free text comments suggested that neurology LOs, in particular the special sense organs such as the eye and ear were not appropriately represented within the Round 1 LOs. Additional LOs from Moxham et al. (2015) were selected and modified by the research team for inclusion into Round 2. Other areas requiring inclusion at this stage were the course and relationships of the oesophagus, the thyroid gland, salivary glands, and tooth anatomy and dentition and the research team wrote these outcomes based on the specific feedback and phrasing suggested by the Delphi panellists. In total an additional 43 new LOs were generated by the research team for inclusion into the second round of the process, accompanying the 79 modified outcomes.

### 3.3 Delphi round two

122 LOs were presented to the Delphi panel in Round 2. Of these, 86 outcomes were accepted, 10 modified (and subsequently included within the final syllabus) and 26 rejected at this stage. (Table 1). Only 52% of nervous system and special sense organ LOs were accepted in this round, with a large number (14 of 42; 33%) rejected at this stage. In contrast 92%, 89% and 81% of alimentary system, reproductive and urinary systems, and musculoskeletal system LOs were accepted in Round 2 (Figure 2). 73 suggestions for modifications were made at this stage by participants, with this smaller number compared with Round 1 reflecting the reduced number of LOs that allowed the option to modify at this second stage.

### 3.4 The core syllabus summaries

#### 3.4.1 Alimentary

Veterinary graduates should be able to describe the structure and functions of the oral cavity, the muscles of mastication and facial expression, the tongue, pharynx and soft palate and the nervous innervation of these. The understanding of nervous innervation should be sufficient to allow the students to describe consequences of nerve injury to these structures and relate nervous pathways to diagnostic tests of reflexes. Graduates should have knowledge of the anatomy of the salivary glands and dentition of a range of relevant species. Tooth structure, growth and eruption should be understood in relation to dental health and tooth extraction. Veterinary graduates should have a topographical understanding of the path of the oesophagus and the functional anatomy involved in the processes of mastication and swallowing. They should have a detailed knowledge of the anatomy of the abdominal wall to allow competent surgical entry into the abdomen. The graduate should have a good knowledge of the topographical anatomy of the abdominal viscera in common veterinary species, sufficient to allow effective palpation, physical examination, and auscultation as well as interpretation of standard diagnostic images. The blood supply to the abdominal viscera should be understood in a clinically relevant context. A graduate should be able to describe the structure and function of major abdominal organs and features and the relevant species differences, including reptiles, fish, and avian species.

#### 3.4.2 Cardiovascular/respiratory

Veterinary graduates should be able to describe the anatomy of and topographical relationships between upper airway structures, especially in the neck region, in the context of presenting respiratory signs, clinical procedures and airway management. They should be able to describe the topography of the paranasal sinuses and guttural pouch (equids) and their relationships with key adjacent structures to a sufficient level to understand normal and abnormal sinus drainage and guttural pouch infection. They should be able to describe the main vasculature within the neck and thorax, especially in relation to blood sampling and injection. The graduate should be able to describe the anatomy of the thoracic wall and diaphragm and have a functional appreciation of their movements during ventilation. They should be able to describe the anatomy of the lower airways especially the position of trachea and lungs, as well as the blood supply, lymphatic drainage and innervation of the lungs. They should be able to describe the boundaries of the mediastinum, and the detailed anatomy and relationships of structures within the mediastinum, in particular the heart and its vessels. The graduate should have sufficient knowledge of the functional anatomy of the heart, its chambers and valves, as well as the lungs, to be able to effectively auscultate the heart and lungs in a range of species and place ECG leads appropriately. They should be able to identify major thoracic structures on diagnostic images. Graduates should be aware of the major developmental changes in the cardiovascular/respiratory systems in the foetus, as well as in non‐mammalian species (reptiles and fish).

#### 3.4.3 Endocrine, lymphoreticular and skin

A veterinary graduate should be able to describe the position, and functional anatomy of the adrenal, thyroid and parathyroid glands as well as their relationships with nearby structures. The graduate should be able to locate the thyroid gland in a patient. They should be able to identify the thymus gland, and describe the location, parts, function, and control of the pituitary gland. A graduate should be able to locate the major superficial (palpable) lymph nodes in different species and have a knowledge of key lymphatic structures and drainage routes in/from the thorax and abdomen, in particular the thoracic duct. A veterinary graduate should be able to describe the structure and function of mammalian, avian and reptilian skin, with specific reference to homeostasis. They should be able to discuss the anatomy of horns as modified skin structures and recount the innervation and blood supply of these structures as well as their relationship to the paranasal sinuses.

#### 3.4.4 Musculoskeletal

A veterinary graduate should be able to describe comparative and functional spinal anatomy, including the structure of spinal joints, intervertebral discs and associated pathologies. They should be able to describe the anatomy and major landmarks of the axial and abaxial skeleton, and identify these on animals and radiographs. Specific attention should be given to those structures associated with common pathologies such as the anconeal/coronoid process of the ulna. They should be able to describe the structure and function of the main thoracic and pelvic limb joints, including their normal motions and the main muscles which create motion and stability. In particular, the anatomy of the hip and stifle joints in canines should be understood in detail, including the role and position of the ligaments of the stifle and the normal/abnormal motions associated with cruciate ligament rupture, patella luxation and hip dysplasia. They should be aware of comparative differences and features such as the passive stay apparatus in equines. A graduate should be able to describe the anatomy of the brachial and lumbosacral plexi with emphasis on the consequences of injury to these regions. Their knowledge of motor and sensory nervous innervation of the limb should be sufficient to relate findings from an orthopaedic examination to any potential neurological cause and to relate the path of a nerve to potential sites/causes of damage. Graduates should be able to describe the clinically relevant blood supply of the limbs in the context of blood sampling and taking a pulse. The graduate should be able to describe the comparative anatomy of the digit across species, including identifying muscles, tendons and ligaments, and, in the equine should describe the innervation and vasculature of this region to allow performance of diagnostic nerve blocks and to obtain a digital pulse. Relevant soft tissue structures such as major tendons in the equine should be able to be identified on ultrasound. Graduates should be able to describe the functional species‐specific anatomical features of feet to include claws and foot pads in small animals, and hooves in ruminants and equids.

#### 3.4.5 Nervous system and special sense organs

A veterinary graduate should be able to describe the anatomy of the skull, including the major foramina (and their contents), and apply this to carrying out local anaesthetic nerve blocks, palpation and interpreting radiographs. They should be able to describe the anatomy of the ventricular system, the production, composition and circulation of cerebrospinal fluid, the blood/CSF barrier, and the anatomical relationships of the meninges as relevant to root compression, the placement of epidural anaesthesia, and CSF sampling. The blood supply to the brain should be understood and the major species differences known in the context of animal welfare during slaughter and the blood/brain barrier. Graduates should be able to describe the anatomy, function and relations of major structures and features of the brain such that this can be applied to the effect of lesions and epilepsy. Graduates should be able to describe the anatomy of the external and internal parts of the eye, the muscles and nerves involved in generating eye movements and the nerves and pathways involved in vision. They should be familiar with the effect of lesions on visual pathways, the corneal, accommodation and pupillary light reflexes. The graduate should be able to describe the anatomy of the motor and sensory nerves to the head and neck, including sympathetic innervation, as relevant to neurological assessment. They should be able to describe the general organisation and function of the autonomic nervous system. They should be able to describe the anatomy and pathways of the cranial nerves, and discuss relevant clinical tests of their integrity. Graduates should be able to describe the functional anatomy of the external, middle and inner ear, auditory tube and its relationship with the guttural pouch in equids. They should be able to describe the general functional and anatomical arrangements of ascending/descending tracts in the spinal cord, the reflex arc and the influence of higher centres on spinal reflexes. This should include providing examples of the biomechanical and muscular effects of lesions to upper and lower motor neurons, the corticospinal tracts and other descending motor pathways, and the effect of lesions on lower motor neuron function in common veterinary species. The anatomical basis for spinal injuries, disc lesions and common peripheral nerve injuries should be able to be described.

#### 3.4.6 Reproductive and urinary

The veterinary graduate should be able to describe the functional anatomy of the external genitalia in common veterinary species and their normal features on clinical examination. They should be able to describe the clinically relevant vascular supply and lymphatic drainage of the testis, and the anatomy of the inguinal ligament and canal, in relation to inguinal hernias and testicular descent. The graduate should be able to describe the comparative and functional anatomy of erectile function and dysfunction (including innervation), emission and ejaculation. They should be able to describe the comparative anatomy and relations of the accessory sex glands especially the canine prostate in the context of rectal examination. A graduate should be able to name the sites of sperm deposition for common veterinary species as applicable to reproductive techniques in practice. Graduates should be able to describe the anatomy of the female reproductive tract and resulting changes with pregnancy (including the anatomy of the placenta). They should be able to identify a functional and regressing corpus luteum on ultrasound/rectal palpation. The graduate should be able to apply topographical pelvic anatomy to clinical (rectal and vaginal) examination in different domestic species and identifying anatomical structures of the pelvis and perineum on ultrasound and x‐ray images. The graduate should be able to describe the anatomy and function of the pelvic diaphragm in relation to continence/prolapse including its innervation. The graduate should be able to describe the structure and comparative anatomy of the mammary gland with reference to animal husbandry practices, disease and milk production. They should be able to describe the relevant anatomy and process of egg formation in avians, methods for sexing birds, and compare mammalian and avian reproductive anatomy with that of reptiles and fish. The veterinary graduate should be able to describe the anatomy and relationships of the bladder, its innervation, dynamic changes when filling and during pregnancy, and the mechanism of micturition. They should be able to describe the anatomy of the urethra in relation to continence and catheterisation. They should be able to describe the position, functional anatomy, and topographical relations of the kidneys and ureters in common veterinary species in a clinically relevant context and describe the vascularisation of the kidney.

### 3.5 The core syllabus

Here we present the Anatomical Society core syllabus for Veterinary Anatomy (Table 2).

**TABLE 2:** Recommended Veterinary anatomy syllabus. Learning outcomes, by body system, accepted after both rounds of the Delphi process.

Learning Outcome	Accepted round
Section 1: Alimentary system	
Use and appreciate standard anatomical directional terms, planes and movements	2
2 Describe the boundaries and major features of the oral cavity and summarise its sensory innervation	1
3 Describe the muscles of mastication and of facial expression, their nerve supply and consequences of injury to their nerve supply	2
4 Identify the major salivary glands of common domestic species	2
5 Describe the anatomy and function of the tongue in common veterinary species, including its motor and sensory innervation and the role of its extrinsic and intrinsic muscles. Explain how hypoglossal nerve injury causes clinical signs	2
6 Describe the anatomy, function and innervation of the muscles of the pharynx and soft palate. Describe the components of the gag reflex and how they are tested.	1
7 Describe the stages of swallowing and the functions of muscles of mastication, tongue, soft palate, pharynx, larynx and oesophagus during swallowing	1
8 Identify the course and describe relationships of the oesophagus with structures of clinical significance in the cervical, thoracic and abdominal regions	2
9 Describe the dentition and dental formula of adults and young in common veterinary species. Describe the anatomy of the tooth in relation to tooth extraction	2
10 Explain the process of tooth eruption and how it differs from tooth growth. Correlate the features of dental anatomy with dietary habits and relate changes in structural appearance to ageing	2
11 Describe the anatomy and innervation of the muscles of the abdominal wall and their significance in surgical approaches to the abdomen. Discuss their functional relationship with the diaphragm and skeleton and their roles in posture, ventilation and voiding of abdominal/thoracic contents	2
12 Describe the topographical anatomy and clinical significance of the abdominal organs in common veterinary species (liver, gall bladder, pancreas, spleen, kidneys, stomach, duodenum, jejunum, ileum)	2
13 Describe the topographical anatomy of the large intestine (caecum, appendix, ascending, transverse, descending and sigmoid parts of the colon) including clinical significance, and species differences for the common veterinary species	2
14 Describe the topographical relationship between abdominal structures palpated during physical examination of common veterinary species, and identify landmarks for auscultation	2
15 Describe the origins, courses and major branches of the abdominal aorta (coeliac artery and its branches, cranial and caudal mesenteric arteries, renal artery, and gonadal arteries). Explain the clinical significance of the blood supply to the abdomen	2
16 Describe the origin and course of the caudal vena cava and its major tributaries	1
17 Describe the position and anatomy of the stomach including sphincters, vascular, and nerve supply and key relations to other abdominal organs. Describe common species differences in the anatomy of the stomach	1
18 Describe the vascular supply of the stomach and spleen and the differences between common veterinary species. Recognise the spleen ultrasonographically	2
19 Outline the organisation and clinical significance of the parietal and visceral peritoneum, the greater and lesser omentum, mesenteries, and peritoneal attachments	2
20 Describe the functional anatomy of the small intestine including the structure, function, location, and blood and nerve supply in common veterinary species	2
21 Describe the duodenum in terms of position within the abdomen, blood and nerve supply, and topographical relationships with other organs, and blood supply relationship to the pancreas	2
22 Describe the positions and functions of the large intestine and the topography in relation to clinical examination, including rectal examination. Describe vascular, lymphatic and nerve supply in common veterinary species, and relate variations in hindgut anatomy in animals with different diets and lifestyles	2
23 Describe the anatomy, topography and function of the caecum in small lagomorphs and rodents, horses and birds	1
24 Describe the position and functional anatomy of the liver and gall bladder, its blood supply and key anatomical locations and differences in liver anatomy between common veterinary species	2
25 Summarise the functional anatomy and clinical significance of the portal vein, the portal venous system and the porto‐systemic anastomoses	2
26 Describe the position and functional anatomy of the gall bladder and biliary tree, including species variations; explain their relations in the abdomen, and the clinical significance of inflammation of the biliary system and biliary stones	2
27 Describe the position, form and blood supply in the pancreas and its ducts, including species differences. Discuss the relationships with other abdominal organs, and clinical significance (e.g. pancreatitis and biliary disease)	2
28 Describe the structure and location of the anal sacs in common veterinary species	2
29 Identify major abdominal structures in standard diagnostic images, e.g.: x‐ray and ultrasound of the abdomen	2
30 Describe the major organs of the gastrointestinal system in reptiles, fish and avian species	1
**Section 2: Cardiovascular/respiratory systems**	
31 Describe the bones and structures of the nasal cavity, in particular the major features of the nasal cavity and the divisions created by the conchae. Describe the clinical importance of its communications and vasculature in relation to common presenting signs and clinical procedures	2
32 Name the paranasal sinuses and identify topographical landmarks for surgical approaches. Describe their relationship to the nasal cavity, teeth, and their sites of drainage in equids	2
33 Explain the clinical significance of the topographical relationship between the trachea, larynx, and other structures of the ventral neck region in relation to airway management	2
34 Identify the landmarks and boundaries of the guttural pouch in equids, including associated nerves and blood vessels and endoscopic access. Describe the appearance of the guttural pouch on radiographic images	2
35 Describe the path of the nasolacrimal duct and recognise its relationship to the nasal cavity and sinuses and the clinical significance of this	2
36 Describe the anatomy of the major vascular structures passing between the neck, and the thorax and forelimb	2
37 Describe the position of the external jugular veins and the surface landmarks that are used for blood sampling and intravenous injection in common veterinary species	2
38 Describe the functional anatomy of the intercostal muscles. Describe a neurovascular bundle in a typical intercostal space, with relation to thoracocentesis	2
39 Describe the attachments and relations of the diaphragm and the structures that pass through it. Explain the movements of the diaphragm and its innervation and pleural coverings	2
40 Describe the anatomy of the joints between the ribs, vertebrae, costal cartilages and sternum. Explain their contribution to the movements of ventilation	1
41 Describe the boundaries of the thoracic inlet and outlet, the structures that pass through them and their relations	1
42 Describe the surface markings of the heart and great vessels, the margins of the pleura, and the lobes and fissures of the lungs in common veterinary species. Explain their clinical relevance	2
43 Identify the major anatomical features of each chamber of the heart and explain their functional significance	1
44 Describe the structure and position of the atrioventricular, pulmonary, and aortic valves and describe their function in the prevention of reflux of blood during the cardiac cycle	1
45 Demonstrate the surface markings of the heart and the position and site of auscultation of its four major valves	1
46 Describe the course of the ascending aorta, the arch of the aorta and the thoracic aorta. Name their major branches and the structures they supply	1
47 Describe the relationship of the pericardium to the heart and relate it to conditions such as cardiac tamponade and pericarditis	2
48 Describe the anatomical course of the spread of electrical excitation through the chambers of the heart in relation to ECG lead placement	2
49 Demonstrate the position of the lungs and areas for auscultation and clinically accessible lung fields in common veterinary species.	1
50 Describe the blood supply, innervation, and venous and lymphatic drainage of the lungs	2
51 Describe the structures in the hilum of the lung and their relationships to each other and the mediastinum	2
52 Describe the foetal circulation system and the functional changes that occur postpartum	2
53 Describe the anatomy of the major groups of lymph nodes in the head and neck, their significance when carrying out a clinical exam, and the potential routes for the spread of infection and malignant disease	2
54 Describe the species adaptations of the respiratory system of reptiles and fish	1
55 Identify major thoracic structures on standard diagnostic images (radiographs and ultrasound)	2
Section 3: Endocrine, lymphoreticular and skin	
56 Describe the divisions of the pituitary into adenohypophysis and neurohypophysis, as well as the control of the pituitary and the general principles of neuroendocrinology	1
57 Describe the position and relations of the adrenal glands and their functional anatomy	1
58 Describe the position, function and anatomy of the thyroid and parathyroid glands, their blood supply and the significance of the courses of the laryngeal nerves. Identify the thyroid gland during a clinical examination	1
59 Identify the thymus in common veterinary species	1
60 Identify palpable lymph nodes in the healthy animal and recognise species differences	1
61 Describe the course and major relations of the thoracic duct. Explain the lymph drainage within the thorax and its clinical significance	1
62 Describe the lymphatic drainage of the stomach, duodenum, small and large intestines and their mesenteries	1
63 Describe the anatomy of the lymph nodes draining the abdominal viscera and their significance in relation to metastatic spread	1
64 Identify the Bursa of Fabricius in avian species	1
65 Describe the structure of mammalian, avian and reptilian skin with specific reference to homeostasis	1
66 Describe the innervation and vascular supply to horns and the interconnection between the paranasal sinuses and horns, specifically with regards to dehorning	2
Section 4: Musculoskeletal system	
67 Identify the atlas, axis, cervical, thoracic, lumbar, sacral, and caudal vertebrae, their projections/processes and explain how these allow identification of vertebra by region. Describe variations in the vertebral formula across common veterinary species	2
68 Describe the regions and functions of the vertebral column, the range of motion of the vertebral column in its entirety and explain the anatomical basis of common spinal injuries/pathologies, e.g. intervertebral disk disease	2
69 Discuss the clinical importance of the principal muscles, ligaments, and surface features to stability and movement of the vertebral column	2
70 Interpret standard diagnostic images of the vertebral column, e.g. X‐rays	2
71 Demonstrate the main anatomical features and surface landmarks of the thoracic vertebrae, ribs and sternum	1
72 Describe the main bones and skeletal landmarks of the thoracic limb of the main veterinary species, especially those that commonly show pathology, e.g. anconeal/coronoid processes of ulna	2
73 Describe the anatomy of the elbow joint. Demonstrate the movements of flexion and extension. Identify the muscles responsible for these movements. Name the main attachments and nerve supply of these muscles	1
74 Describe the functional anatomy of the radioulnar joint with respect to supination and pronation. Relate anatomical differences across species in this region to the range of possible limb movements	2
75 Describe the anatomy and motions of the carpus across common veterinary species; name and identify the muscle groups responsible for these movements. Describe the relative positions of tendons, vessels, and nerves in the region of the carpus in relation to common injuries	2
76 Describe the anatomy of the digit(s) in common veterinary species. Describe the position, function, and nerve supply of the muscles and tendons involved in the movements of the digit(s). Compare the relative position of these structures across species and note any specific species adaptations (e.g. Interosseus mm. vs. suspensory ligament)	2
77 Describe the neurovascular structures lying in close relation to the bones and joints of the thoracic limb which are at risk of injury following fracture or dislocation. Predict what the functional effects of such injury might be	1
78 Identify the location of the brachial plexus; describe the spinal nerves that contribute to the brachial plexus, and the major thoracic limb nerves arising from it. Describe the functional significance of the brachial plexus and explain how you would recognise a brachial plexus injury	2
79 Describe the sensory and motor distribution of the axillary, radial, musculocutaneous, median, and ulnar nerves and state the major muscle groups these nerves supply. Describe the consequences of injury to these nerves and how to test their functional integrity	2
80 Describe the anatomical basis of assessment of cutaneous sensation in the thoracic limb	1
81 Describe the course of the main veins of the thoracic limb. Identify the common sites of venous access and describe their key anatomical relations	1
82 Describe the structures comprising the passive stay apparatus in the forelimb and explain the function of this apparatus in equids	1
83 Describe the position and function of retinacula and tendon sheaths in order to explain injuries and the spread of infection in tendon sheaths	1
84 Describe the bony, tendinous and ligamentous structures of the equine distal limb and recognise these structures on x‐ray and ultrasound	1
85 Describe the species‐specific anatomical features of the foot to include claws and foot pads in small animals, and hoof structure in ruminants and equids. Relate the structure of these adaptations to their function and susceptibility to injury	1
86 Interpret standard diagnostic images, e.g. X‐rays and ultrasound of the thoracic limb, and identify key structures of the thoracic limb	2
87 Describe the osteology and surface landmarks of the pelvis (ilium, ischium and pubis), femur, tibia, fibula tarsus, metatarsus and digit(s). Demonstrate their palpable and imaging landmarks. Explain how the bones, joints and related structures are vulnerable to damage and what the consequences of such damage could be	1
88 Describe the course of the major arteries and veins that supply the pelvic limb and the main sites for venous access in common veterinary species	2
89 Describe the boundaries and contents of the femoral triangle with particular regard to taking an arterial pulse	1
90 Describe the locations at which the femoral, dorsal pedal and digital arterial pulses can be palpated in common veterinary species	2
91 Describe the basic organisation of the lumbosacral plexus including its spinal roots and the major branches arising. Describe the functional significance of the lumbosacral plexus and explain how you would recognise a lumbosacral plexus injury	2
92 Describe the origin, course, and function of the femoral, obturator, sciatic (ischiadic), tibial and common fibular (peroneal) nerves; summarise the muscle groups that each supplies, as well as their sensory distribution	2
93 Describe the anatomy of the gluteal region and the course of the sciatic nerve through it. Be aware of potential risk of damage to the sciatic nerve when giving intramuscular injections	2
94 Describe the nervous innervation of the equine distal limb particularly in relation to the standard sites for diagnostic nerve blocks	1
95 Describe the anatomy and movements of the hip joint. Summarise the muscles responsible for these movements, their innervation and attachments. Describe the importance of hip joint anatomy in relation to hip dysplasia in dogs	1
96 Describe the structures responsible for stability of the hip joint	1
97 Describe the anatomy and movements of the stifle joint. Summarise the muscles responsible for these movements, their innervation and main attachments	1
98 Identify the factors responsible for maintaining the stability of the stifle joint. Describe the locking mechanism in horses. Explain the anatomical basis of tests (including imaging/radiographs) that assess the integrity of the cruciate ligaments and patella luxation in small animals	2
99 Describe the anatomy and movements of the tarsus and tarsal joints. Summarise the muscles responsible for these movements, their innervation and their attachments.	1
100 Interpret standard diagnostic images of the pelvic limb, e.g. X‐ray and ultrasound, recognising common pelvic limb structures and landmarks	2
Section 5: Nervous system and special sense organs	
101 Describe the anatomy of the skull and the position of the palpable and radiographic landmarks of the major bones of the skull in common veterinary species	2
102 Identify the major foramina of the skull externally, as relevant for local anaesthetic nerve blocks, and list the structure(s) that each transmits	2
103 Describe the basic anatomy of the ventricular system and the circulation and drainage of cerebrospinal fluid	2
104 Describe the structure, function, formation and absorption of cerebrospinal fluid.	1
105 Describe the blood supply to the brain including the major species variations. Describe the relevance of these variations to animal welfare during slaughter	1
106 Describe the structure and functional significance of the Blood–brain and blood‐CSF barriers	1
107 Describe the location of the medulla oblongata, its major anatomical features and its relationship to other components of the brainstem, both anatomically and functionally.	1
108 Describe the gross anatomical appearance of the pons and relate the pons to the other components of the brainstem	1
109 Describe the gross appearance of the cerebellum and its relationship to other regions of the brainstem	1
110 Describe the location and relationships of the thalamus and hypothalamus, the general functions of the thalamus/hypothalamus, and efferent and afferent connections	2
111 Describe the location and relationships of the optic chiasma and pituitary gland with the rest of the diencephalon	2
112 Describe the structural components of the cerebral hemispheres, including the organisation into sulci and gyri of the cerebral cortex, the lobes of the cerebral hemisphere, and lateral ventricles	2
113 Describe the location and function of the areas of the cerebral cortex, including: frontal lobe, parietal lobe, occipital lobe, temporal lobe, the vestibular area, and the association cortex	2
114 Describe the relationship of lesions to the cerebral cortex and epilepsy	2
115 Outline the location and function of the limbic system and hippocampal formation	2
116 Describe the anatomy of the eyelid, nictating membrane, conjunctiva, and lacrimal gland. Explain their importance for the maintenance of corneal integrity	2
117 Describe the boundaries of the orbit and globe of the eye, and the location, actions, and nerve supply of the intrinsic and extraocular muscles. Explain the consequences of injury to their nerve supply	2
118 Describe neurons of the visual pathway and explain the process of binocular vision. Describe the principles behind the species differences in bino‐ versus mono‐cular vision	2
119 Describe the autonomic innervation of the eye, upper eyelid, and iris, to include pupillary light reflexes, and the accommodation reflex	2
120 Describe the corneal reflex and its clinical significance	2
121 Explain the resultant effects of lesions within the visual pathway	2
122 Describe the anatomy of the motor and sensory nerves to the head and neck and apply this knowledge to a neurological assessment of the cranial and upper cervical spinal nerves	2
123 Describe the sympathetic innervation of the head and neck including the features and main causes of Horner's syndrome	2
124 Describe the anatomy and central connections of the cranial nerves and discuss clinical tests for their examination	2
125 Describe the anatomy of the sensory and motor components of the trigeminal nerve, including how their integrity is tested clinically	2
126 Describe the functional anatomy of the auricle, external auditory meatus, tympanic membrane, auditory ossicles, and auditory tube as well as its relationship with the guttural pouch in equids	2
127 Describe the radiographic appearances of the intracranial cavity and the vertebral column	2
128 Describe the anatomical relationships of the meninges to the spinal cord and dorsal and ventral nerve roots, particularly in relation to root compression, the placement of epidural anaesthesia and CSF sampling	2
129 Describe the general functional and anatomical arrangements of ascending/descending tracts in the spinal cord	2
130 Describe the reflex arc and the influence of higher centres on spinal reflexes, upper motor neuron lesions, and lesions of the corticospinal tracts (‘pyramidal’ tracts) and of descending motor pathways other than the corticospinal tracts (the ‘extrapyramidal’ tracts	2
131 Describe and give examples of the effects of lesions to lower motor neuron function	2
132 Describe how lesions to the nervous system manifest clinically as biomechanical and muscular symptoms in common veterinary species	2
133 Describe the effects of complete transection of the spinal cord	2
134 Describe the anatomical bases (nerve root or peripheral nerve) for loss of limb movements and reflexes resulting from spinal injuries, disc lesions, and common peripheral nerve injuries	2
135 Describe the general organisation and function of the autonomic nervous system, to include sympathetic efferent nerve fibres (sympathetic outflow), sympathetic afferent nerve fibres, sympathetic trunks and ganglia, parasympathetic efferent nerve fibres (craniosacral outflow), parasympathetic afferent nerve fibres, the major autonomic plexuses, parasympathetic autonomic ganglia.	1
Section 6: Reproductive and urinary systems	
136 Describe the anatomy of the inguinal ligament and inguinal canal in the male and female	1
137 Explain the contents of the canal and how inguinal hernias develop, including the anatomy and clinical presentation of such hernias	1
138 Describe the anatomy of the penis, scrotum, testis and epididymis in common veterinary species and their normal features on clinical examination. Explain the significance of the vascular supply and lymphatic drainage of the testis in relation to castration and torsion/varicocele	1
139 Describe the innervation of, and mechanisms involved in, the erection of cavernous tissue in males and females with particular relevance to paraphimosis. Describe the mechanisms involved in emission and ejaculation in the male and the process of erection in the fibroelastic penis	2
140 Name the sites of sperm deposition for common veterinary species and the applicability to reproductive techniques in practice	2
141 Describe the structure and course of the spermatic cord and ductus (vas) deferens	1
142 Describe the anatomy and relations of the accessory sex glands in common veterinary species	1
143 Describe the normal form of the canine prostate when examined per rectum and how this changes in relation to hypertrophy, malignancy, infection or inflammation	2
144 Describe the anatomy and relations of the ovary, uterine tubes, uterus, cervix and vagina, including their peritoneal coverings in common veterinary species. Describe the changes that occur in the uterus and cervix with pregnancy	1
145 Identify a functional and regressing corpus luteum. Recognise these stages on ultrasound in common veterinary species and on rectal palpation in large ruminants and horses	2
146 Describe what structures are normally palpable on rectal and vaginal examination in different domestic species and how to undertake these examinations	2
147 Describe the anatomy and functional importance of the pelvic diaphragm and its clinical significance, e.g. in relation to continence/prolapse	2
148 Describe the origin, course and distribution of the pudendal nerves and the sites of pudendal nerve block	1
149 Describe the anatomy of the mammary gland including its neurovascular supply and lymphatic drainage (with reference to clinical relevance to disease, metastatic spread and milk production)	1
150 Compare the position and arrangement/number of mammary glands and ducts/teats in common veterinary species and discuss the clinical significance of these differences	2
151 Outline the main anatomical features of the placenta in common veterinary species	1
152 Describe the process of egg formation and identify where each step takes place in the avian reproductive tract	1
153 Describe the methods for sexing birds	1
154 Describe patterns of reptile and fish reproductive anatomy	1
155 Describe the anatomy of the bladder, its base and ureteric openings and its relationship to the overlying peritoneum. Explain how the position of the bladder changes with filling and during pregnancy	1
156 Describe the anatomy of the urethra; explain the anatomy of its different parts in males and females in relation to continence and catheterisation	1
157 Describe the innervation of the bladder, its sphincters and the mechanism of micturition	1
158 Describe the position, functional anatomy and topographical relations of the kidneys and ureters in common veterinary species. Discuss the clinical significance of renal and ureteric anatomy in particular in relation to urinary stones and ovariohysterectomy	2
159 Describe the vascularisation of the kidney	1
160 Interpret and identify anatomical structures on standard diagnostic images (ultrasound and x‐ray) of the pelvis and perineum	2

### 3.6 Contextual comments

The rationale for modification and rejection of LOs is summarised in Table 3. Reasons cited for modification were grouped into three main categories, each with several sub‐categories. The first category, Typography, includes suggestions in relation to rephrasing, spelling and grammar, or referred to appropriateness within the taxonomy of verbs. Maintaining consistency throughout the LOs was also identified as a reason for modification. The second category, Emphasis, reflects perceptions that LOs should focus on Day One Competences or that the clinical relevance or functional anatomy should be highlighted. This category also includes references to anatomical variation across species, or that relevant species be highlighted within the LO. The final category, Level of information, includes comments suggesting less detail be included, or that specific information form part of the LO.

**TABLE 3:** Analysis of free text comments detailing rationale for modifications made to learning outcomes (LO).

Category	Sub‐category	Description	Example comments
Typography	Consistency	Suggestions to make LO more in line with others by including more/similar topics or by rewording LO	*Some inconsistencies for when diagnostic imaging is included—*i.e. *‘identify the spleen on ultrasound’ could be covered by ILO26 or could argue it inclusion into each individual ILO* i.e. *recognising the gall bladder. ILO 13 mentions abdominal organs but does not include urinary bladder and reproductive organs*
	Terminology and grammar	Identification of typographical errors, poorly phrased LOs, or where human anatomy specific terms or content were included	*Replace ‘glands’ with ‘sacs’* *Origin/insertion more commonly used for attachments*
	Sequence	LO included in wrong body system or out of sequence	*Outcome fine but should this be listed in the urinary section rather than the repro?*
	Repetition	LO or part LO covered elsewhere, suggestions to combine LOs, identification of repetition or duplication	*Maybe No. 70 and No. 71 could be combined?* *Same as ILO 138*
	Separate out	Where an LO should be split into more LOs	*This learning outcome is composed of two long objectives, that should rather be separated*
	Taxonomy	Suggested changes to taxonomy of verbs—difficult to test certain outcomes	*How is this demonstrable? Maybe ‘describe the differences…’*
Emphasis	Day One Competences	Comments reflecting the need to focus the LO on outcomes relevant for a new graduate. (common for imaging techniques)	*New grad focus—radiography/ultrasound* *Day 1 competent vets should be able to identify that there is a neural problem in order to communicate effectively with specialists*
	Functional anatomy	Change emphasis to functional anatomy	*Changes to the ovaries in follicular and luteal phases*
	Clinical relevance	Clinical significance should be included or emphasised	*There should be an emphasis on surgical anatomy* *Explain the clinical significance of these structures, especial with regard to ovariohysterectomy and pregnancy diagnosis*. *Describe the innervation and vascular supply to horns and the interconnection between sinuses and horns, with the implications for dehorning*
	Relevant species	Reword so that relevant species are covered. Including naming specific species or ‘mammals’ or ‘common veterinary/domestic’ species Includes change of emphasis from human	*In common veterinary species to recognise differences* *Vocal cord position and tension could arguably be omitted as less important in domestic species than human anatomy*.
	Species variation	Where specific reference should be made to species differences	*Demonstrate the location of the sacrotuberous ligament in small animals and the sacrosciatic ligament in large animals*.
	Not anatomy	LO that cover topics other than anatomy, e.g. pathology, physiology, clinical examination. Suggestions to focus on anatomy	*If this is purely an anatomical checklist there should be no requirement to understand pathologies*
Level of information	Abnormalities/dysfunction	Suggestions that abnormalities/pathologies, and processes such as inflammation should be included. Recognition of dysfunction and injuries in relation to nerves	*Explain how dysfunction results in clinical signs and the anatomical pathology present with portosystemic shunts* (*intra and extra hepatic*)
	Blood and nerve supply	Blood and or nerve supply should also be included	*Also coccygeal artery* (*farm*)
	Histological structure	Suggestions that histology should be included	*Gross anatomical and histological differences between species*
	More specific	Include more specific information	*I would suggest modification to be more specific about relationships to what*. (*Particularly if specific structures are clinically important* e.g. *trachea, diaphragm…?*)
	Remove detail	Suggestions for modifications which remove parts of LOs or reference to specific structures	*Remove: “Explain how these are linked together by the intrinsic and extrinsic laryngeal membranes* *I am not sure that detailed recall of individual spinal tracts is essential but general principles are needed*

Due to the small number of participants selected for this study, and the multiple opportunities provided for comment, counts and magnitude coding were not carried out as part of data analysis. However, the greatest number of comments referred to two main points: the need to reduce the level of detail presented, and the need to focus on topics of relevance to the day one graduate.

## 4 DISCUSSION

The aim of this study was to produce an evidence‐based syllabus comprised of the anatomy outcomes that would need to be achieved by a student by the end of an undergraduate veterinary medicine degree. The LOs presented are the product of a consensus‐based process which utilised the expertise of a panel of experienced anatomy educators teaching on veterinary medicine programmes. The syllabus aims not to be prescriptive, since individual veterinary schools will inherently take their own independent approaches to curriculum design and teaching methods, as appropriate to their local context. However, as a tool for anatomy educators and course leaders the syllabus provides a starting point for beginning to answer the challenging question of ‘What anatomy knowledge does a veterinary graduate need?’

The level of detail and depth of knowledge required in anatomy curricula is much debated (Sugand et al., 2010). Panel member comments in this study suggested that the level of detail required for an individual LO to be meaningful will vary depending on the specific topic to be delivered, and many comments called for a reduction in the detail included in some LOs. Reducing detail also results in a reduction in the volume of information to be learned. Excessive quantity and detail can overburden students and can encourage superficial learning approaches (Cake, 2006; May & Silva‐Fletcher, 2015), understood to negatively affect long‐term knowledge retention (Ward & Walker, 2008). Further comments pertaining to clinical emphasis, and a focus on Day One Competences similarly reflect broader discussions within veterinary curricula.

The importance of an outcomes based veterinary curriculum has been emphasised by May (2015) and others (Cobb et al., 2015; Davis, 2003; Matthew et al., 2020). The Royal College of Veterinary Surgeons requirements (The Royal College of Veterinary Surgeons, 2022) are necessarily broad due to the variety of career options available to a newly graduated veterinary surgeon. Defining anatomically relevant LOs for a curriculum based on Day One Competences therefore is challenging. Additionally, on a global scale, accrediting bodies from different regions each have differing requirements, and so for curriculum leaders aiming to satisfy the requirements of multiple Professional Statutory and Regulatory Bodies, aligning curricula to the required graduate outcomes will continue to be a challenge. As a fundamental science, anatomy underpins and can be found inherently interwoven within multiple graduate outcomes and competencies, from the explicit (carrying out a post‐mortem examination; performing simple elective surgeries) to the more subtle (e.g. the use of correct anatomical terminology when ‘communicating clearly’ or ‘accurately preparing a case report’).

The potential roles and responsibilities of a veterinarian continue to evolve, reflecting technological advances and societal change (Gibbs & Gibbs, 2013; Ruston et al., 2016) and so it will never be possible to define a syllabus which contains all the anatomical knowledge that will be required during an individual's veterinary career. Even for those entering first opinion clinical practice, it is impossible to predict what cases, pathologies, or surgeries a veterinary graduate will encounter on day one, within the first year of graduation and at later stages of their careers. Similarly, within first opinion clinical practice the requirements and uses of anatomical knowledge vary considerably between veterinarians of differing species specialities (Homfray et al., 2022). For this reason, this syllabus only aims to suggest a fundamental core knowledge, which provides sufficient anatomical grounding and appreciation for safe practice of Day One Competences, onto which a veterinarian can build their working repertoire of anatomical knowledge that befits their specific role and experiences (Wheble & Channon, 2021). We requested that our Delphi panel consider the core knowledge of a newly qualified veterinarian in frame as they made their judgements about each LO. Nevertheless, we did not include clinical qualification, or activity as inclusion criteria when selecting the panel, and did not collect information regarding the clinical roles and responsibilities of our participants. Rather, the inclusion criteria for the panel reflected the value placed on understanding and experience of teaching, learning and assessment in undergraduate veterinary education. Further work involving participants with principally clinical roles, particularly those in first opinion practice, might help to further refine and streamline the newly developed syllabus.

## 5 CONCLUSION

Determining the level of anatomical knowledge a new graduate veterinary surgeon needs for competent practice is challenging. Veterinary graduates can enter a broad range of roles across species and specialities, and the profession continues to evolve. A Delphi method can be a valuable tool in establishing a consensus on the content of a veterinary anatomy curriculum, and the curriculum presented as a result of this process can provide a useful structure for curriculum planners worldwide.

## AUTHOR CONTRIBUTIONS

Concept Sarah B. Channon; Study Design Sarah B. Channon, Erica Gummery; Acquisition of data Sarah B. Channon, Erica Gummery, Miren Singh; Data analysis/interpretation Sarah B. Channon, Erica Gummery, Miren Singh; Drafting of the manuscript Sarah B. Channon, Erica Gummery, Miren Singh; Critical revision of the manuscript and approval of the article Sarah B. Channon, Erica Gummery, Miren Singh.

## Data Availability

The data that support the findings of this study are available from the corresponding author upon reasonable request.
